# Supplementary material for: The Role of Wildlife and Pests in the Transmission of Pathogenic Agents to Domestic Pigs: A Systematic Review
Source: Animals (Basel). 2023 May 31;13(11):1830. doi: 10.3390/ani13111830 (PMC10251848; doi:10.3390/ani13111830)
Supplement: Supplementary file 1 [file animals-13-01830-s001.zip › animals-2382781-supplementary.pdf]

Supplementary Table S1: Preferred Reporting Items for Systematic reviews and Meta-Analyses extension for Scoping Reviews (PRISMA-ScR) Checklist [130]

Section and Topic	Item #	Checklist item<br>**Preferred Reporting Items for Systematic reviews and Meta-Analyses (PRISMA-ScR) Checklist**	Location where item is reported
**TITLE**			
Title	1	Identify the report as a systematic review.	1
**ABSTRACT**			
Abstract	2	See the PRISMA 2020 for Abstracts checklist.	1
**INTRODUCTION**			
Rationale	3	Describe the rationale for the review in the context of existing knowledge.	Introduction
Objectives	4	Provide an explicit statement of the objective(s) or question(s) the review addresses.	Introduction
**METHODS**			
Eligibility criteria	5	Specify the inclusion and exclusion criteria for the review and how studies were grouped for the syntheses.	Table 1
Information sources	6	Specify all databases, registers, websites, organisations, reference lists and other sources searched or consulted to identify studies. Specify the date when each source was last searched or consulted.	Materials and methods
Search strategy	7	Present the full search strategies for all databases, registers and websites, including any filters and limits used.	Figure 1, Table 1. Provided as supplementary material (S2)
Selection process	8	Specify the methods used to decide whether a study met the inclusion criteria of the review, including how many reviewers screened each record and each report retrieved, whether they worked independently, and if applicable, details of automation tools used in the process.	Figure 2, Material and Methods
Data collection process	9	Specify the methods used to collect data from reports, including how many reviewers collected data from each report, whether they worked independently, any processes for obtaining or confirming data from study investigators, and if applicable, details of automation tools used in the process.	Material and Methods
Data items	10a	List and define all outcomes for which data were sought. Specify whether all results that were compatible with each outcome domain in each study were sought (e.g. for all measures, time points, analyses), and if not, the methods used to decide which results to collect.	Material and Methods. Also, details have been described in review protocol
	10b	List and define all other variables for which data were sought (e.g. participant and intervention characteristics, funding sources). Describe any assumptions made about any missing or unclear information.	Material and Methods, Limitation. Also, details have been described in review protocol

Section and Topic	Item #	Checklist item<br>Preferred Reporting Items for Systematic reviews and Meta-Analyses (PRISMA-ScR) Checklist	Location where item is reported
Study risk of bias assessment	11	Specify the methods used to assess risk of bias in the included studies, including details of the tool(s) used, how many reviewers assessed each study and whether they worked independently, and if applicable, details of automation tools used in the process.	Material and Methods
Effect measures	12	Specify for each outcome the effect measure(s) (e.g. risk ratio, mean difference) used in the synthesis or presentation of results.	Results, Figure 3, Table 2
Synthesis methods	13a	Describe the processes used to decide which studies were eligible for each synthesis (e.g. tabulating the study intervention characteristics and comparing against the planned groups for each synthesis (item #5)).	Table 1, Figure 2<br>Also, details have been described in review protocol
	13b	Describe any methods required to prepare the data for presentation or synthesis, such as handling of missing summary statistics, or data conversions.	NA
	13c	Describe any methods used to tabulate or visually display results of individual studies and syntheses.	NA
	13d	Describe any methods used to synthesize results and provide a rationale for the choice(s). If meta-analysis was performed, describe the model(s), method(s) to identify the presence and extent of statistical heterogeneity, and software package(s) used.	Material and Methods
	13e	Describe any methods used to explore possible causes of heterogeneity among study results (e.g. subgroup analysis, meta-regression).	NA
	13f	Describe any sensitivity analyses conducted to assess robustness of the synthesized results.	NA
Reporting bias assessment	14	Describe any methods used to assess risk of bias due to missing results in a synthesis (arising from reporting biases).	Material and Methods
Certainty assessment	15	Describe any methods used to assess certainty (or confidence) in the body of evidence for an outcome.	NA
**RESULTS**			
Study selection	16a	Describe the results of the search and selection process, from the number of records identified in the search to the number of studies included in the review, ideally using a flow diagram.	Table 1, Figure 2
	16b	Cite studies that might appear to meet the inclusion criteria, but which were excluded, and explain why they were excluded.	Mat and methods, Limitations of this review. The detail information can be provided on request
Study characteristics	17	Cite each included study and present its characteristics.	Results, Table 2
Risk of bias in studies	18	Present assessments of risk of bias for each included study.	NA
Results of individual studies	19	For all outcomes, present, for each study: (a) summary statistics for each group (where appropriate) and (b) an effect estimate and its precision (e.g. confidence/credible interval), ideally using structured tables or plots.	NA
Results of	20a	For each synthesis, briefly summarise the characteristics and risk of bias among contributing studies.	NA

Section and Topic	Item #	Checklist item	Location where item is reported
syntheses	20b	Present results of all statistical syntheses conducted. If meta-analysis was done, present for each the summary estimate and its precision (e.g., confidence/credible interval) and measures of statistical heterogeneity. If comparing groups, describe the direction of the effect.	NA
	20c	Present results of all investigations of possible causes of heterogeneity among study results.	Results
	20d	Present results of all sensitivity analyses conducted to assess the robustness of the synthesized results.	NA
Reporting biases	21	Present assessments of risk of bias due to missing results (arising from reporting biases) for each synthesis assessed.	Limitations of this review
Certainty of evidence	22	Present assessments of certainty (or confidence) in the body of evidence for each outcome assessed.	Limitations of this review
**DISCUSSION**			
Discussion	23a	Provide a general interpretation of the results in the context of other evidence.	Discussion
	23b	Discuss any limitations of the evidence included in the review.	Limitations of this review
	23c	Discuss any limitations of the review processes used.	Limitations of this review
	23d	Discuss implications of the results for practice, policy, and future research.	Discussion
**OTHER INFORMATION**			
Registration and protocol	24a	Provide registration information for the review, including register name and registration number, or state that the review was not registered.	NA
	24b	Indicate where the review protocol can be accessed, or state that a protocol was not prepared.	Review protocol was prepared and is available upon request
	24c	Describe and explain any amendments to information provided at registration or in the protocol.	NA
Support	25	Describe sources of financial or non-financial support for the review, and the role of the funders or sponsors in the review.	Ghent University (Belgium)
Competing interests	26	Declare any competing interests of review authors.	Conflicts of Interest
Availability of data, code and other materials	27	Report which of the following are publicly available and where they can be found: template data collection forms; data extracted from included studies; data used for all analyses; analytic code; any other materials used in the review.	References

From: Page MJ, McKenzie JE, Bossuyt PM, Boutron I, Hoffmann TC, Mulrow CD, et al. The PRISMA 2020 statement: an updated guideline for reporting systematic reviews. BMJ 2021;372:n71. doi: 10.1136/bmj.n71

For more information, visit: http://www.prisma-statement.org/

Supplementary Table S2: Detailed search strings used in each database

A systematic review on *"What is the role of wildlife species and pests on the transmission of infectious pathogens in pig farms in European region?"*

Subject	Key string
**Domestic pigs**	pig OR pigs OR "farm pig" OR "farm pigs" OR swine OR sow OR porcine OR hog OR "commercial pigs" OR "commercial pigs farming" OR "commercial swine industry" OR "commercial swine production" OR "intensive pig farming" OR "intensive pig farms" OR "intensive pig production" OR "pig factory farming" OR "pig farming" OR "free range pig farming" OR "free-ranging pig holding" OR "free range pigs" OR "open air pig farming" OR "outdoor pig production"
**Wildlife species and pests**	"wild pig" OR "wild pigs" OR "wild boar" OR "wild boars" OR "wild swine" OR "wild hog" OR "feral pig" OR "feral pigs" OR "feral hog" OR "feral hogs" OR "Sus scrofa" OR "Old World swine" OR "feral swine" OR "wild birds" OR "waterfowl" OR "aquatic birds" OR "pigeons" OR "starlings" OR "ducks" OR "homeless dogs" OR "stray dogs" OR "stray dog" OR "cats" OR "rodents" OR "rats" OR "mice" OR "house mouse" OR "Mus musculus" OR "brown rat*" OR "Norwegian rat" OR "Rattus norvegicus" OR "feral rodents" OR "black rats" OR "house mice" OR "barn rats" OR "wild rodents" OR "rodents on farms" OR "mice" OR "deer" OR "red deer" OR "fallow deer" OR "badgers" OR "hare" OR "European hare" OR "wild hares" OR "North European hare" OR "European brown hare" OR "marten" OR "racoon dogs" OR "moles" OR "rabbit " OR "hamster" OR "ferret" OR "flies" OR "wolves" OR ticks
**Spread, vectors, routes of transmission**	"spread of infection" OR "spreading of microbes" OR "spread of infectious agents" OR "spread of pathogens" OR "movement of pathogens" OR "transfer of viruses" OR "transfer of an infectious agent" OR "transmission of pathogens" OR "transmission of pathogens to pigs farms" OR "transmission of infectious pathogens" OR transmission OR "chain of Infection" OR "circulation of infection" OR "routes of infection" OR "vectors of infection" OR vectors OR "transmitted by vectors" OR "transmission of diseases" OR "carries a disease" OR "carry out a disease" OR "biological vector" OR "biological vectors" OR "disease vector" OR "disease vectors" OR "mechanical vector" OR "mechanical vectors" OR "direct contact" OR fomites OR "vector borne" OR "indirect contact" OR foodborne OR "vehicle borne"
**Diseases (Pathogens)**	"African swine fever" OR ASF OR ASFV OR "Classical swinefFever" OR CSF OR "hog cholera" OR "pig plague" OR Salmone* OR "Salmonella spp." OR Salmonella OR "Salmonella Typhimurium" OR "Aujeszky's disease" OR Pseudorabies OR PRV OR "Brucella suis" OR "swine brucellosis" OR "Brucellossis in pigs" OR Brucella OR "Brucella abortus" OR Influenza OR "Porcine Influenza" OR "influenza type A" OR "swine influenza virus" OR "FMD" OR "Foot-and-Mouth Disease" OR "FMDV" OR "Leptospirosis" OR "Weil's disease" OR "Leptospira interrogans" OR "Leptospira borgpetersenі" OR "Leptospira bratislava" OR "Leptospira icterohaemorrhagiae" OR Trichinellosis OR "Trichinella spiralis" OR "Trichinella britova" OR "Trichinella spp" OR Trichinella OR

	HEV OR "Hepatitis E" OR "Rabies virus" OR RABV OR Tuberculosis OR "Mycobacterium avium" OR "avian tuberculosis" OR "Mycobacterium tuberculosis" OR "Mycobacterium bovis" OR "Transmissible gastroenteritis" OR TGE OR "Swine Erysipelas" OR "Erysipelothrix rhusiopathiae" OR Erysipela OR PED OR "Porcine epidemic diarrhoea virus" OR "Porcine epidemic diarrhea" OR PRRS OR "Porcine reproductive and respiratory syndrome " OR "swine dysentery" OR "Brachyspira hyodysenteriae" OR "B. pilosicoli" OR Encephalomyocarditis OR EMCV OR "virus of encephalomyocarditis" OR "atrophic rhinitis" OR "Pasteurella multocida" OR "B. bronchiseptica" OR "enzootic pneumonia" OR "Mycoplasmal pneumonia" OR "Mycoplasma hyopneumoniae" OR dermatophytes OR "Trichophytum mentagrophytes" OR "Streptococcal infections" OR "Streptococcus suis" OR "Streptococcus equisimilis" OR "Streptococcus porcinus" OR "porcine proliferative enteropathy" OR "Lawsonia intracellularis bacterium" OR "yersiniosis" OR "Yersinia pseudotuberculosis" OR "Y. enterocolitica" OR campylobacteriosis OR "Campylobacter jejuni" OR "Campylobacter hyointestinalis" OR "Campylobacter mucosalis" OR "Campylobacter coli" OR "Actinobacillus equuli" OR "Actinobacillus suis" OR Actinobacillosis OR Toxoplasmosis OR "Toxoplasma gondii" OR "Cryptosporidium suis" OR Cryptosporidiosis OR "Cryptosporidium parvum" OR giardiosis OR "Giardia intestinalis" OR "Methicillin-resistant Staphylococcus aureus infection" OR "staphylococcus aureus" OR MRSA OR Bordetellosis OR " Bordetella bronchiseptica" OR "Haemophilus parasuis" OR Colibacillosis OR "Escherichia coli" OR "Glasser disease" OR "Glaesserella parasuis" OR "Porcine circovirosis type 2" OR PCV2 OR "Swine vesicular disease" OR "Swine vesicular disease virus" OR EVP
**Area**	Europe OR "European countries" OR "EU countries" OR "EU country" OR "EU member" OR "non-EU country" OR Albania OR Andorra OR Austria OR Belarus OR Belgium OR "Bosnia and Herzegovina" OR Bulgaria OR Croatia OR "Republic of Cyprus" OR "Czech Republic" OR Denmark OR Estonia OR Finland OR France OR Germany OR Greece OR "Holy See" OR Hungary OR Iceland OR Ireland OR Italy OR Latvia OR Liechtenstein OR Lithuania OR Luxembourg OR Malta OR Moldova OR Monaco OR Montenegro OR Netherlands OR "North Macedonia" OR Norway OR Poland OR Portugal OR Romania OR "San Marino" OR Serbia OR Slovakia OR Slovenia OR Spain OR Sweden OR Switzerland OR Ukraine OR "United Kingdom"
**Time frame**	01.01.2010-31.12.2022

Pubmed

(pig*[Title/Abstract] OR farm pig[Title/Abstract] OR farm pigs[Title/Abstract] OR swine[Title/Abstract] OR sow[Title/Abstract] OR porcine[Title/Abstract] OR hog[Title/Abstract] OR commercial pigs[Title/Abstract] OR commercial pigs farming[Title/Abstract] OR commercial swine industry[Title/Abstract] OR commercial swine production[Title/Abstract] OR intensive pig farming[Title/Abstract] OR

intensive pig farms[Title/Abstract] OR intensive pig production[Title/Abstract] OR pig factory farming[Title/Abstract] OR pig farming[Title/Abstract] OR free range pig farming[Title/Abstract] OR free-ranging pig holding[Title/Abstract] OR free range pigs[Title/Abstract]) AND (wild pig[Title/Abstract] OR wild pigs[Title/Abstract] OR wild boar*[Title/Abstract] OR wild swine[Title/Abstract] OR wild hog[Title/Abstract] OR feral pig*[Title/Abstract] OR feral hog*[Title/Abstract] OR Sus scrofa[Title/Abstract] OR Old World swine[Title/Abstract] OR feral swine[Title/Abstract] OR wild birds[Title/Abstract] OR aquatic birds[Title/Abstract] OR waterfowl [Title/Abstract] OR pigeons[Title/Abstract] OR starlings[Title/Abstract] OR ducks [Title/Abstract] OR homeless dogs[Title/Abstract] OR stray dog*[Title/Abstract] OR cats[Title/Abstract] OR waterfowl[Title/Abstract] OR pigeons[Title/Abstract] OR rodents [Title/Abstract] OR rats[Title/Abstract] OR mice[Title/Abstract] OR house mouse[Title/Abstract] OR Mus musculus[Title/Abstract] OR brown rat*[Title/Abstract] OR Norwegian rat[Title/Abstract] OR Rattus norvegicus[Title/Abstract] OR feral rodents[Title/Abstract] OR black rats[Title/Abstract] OR house mice[Title/Abstract] OR barn rats[Title/Abstract] OR wild rodents[Title/Abstract] OR feral rodents[Title/Abstract] OR rodents on farms[Title/Abstract] OR mice[Title/Abstract] OR deer[Title/Abstract] OR red deer[Title/Abstract] OR fallow deer[Title/Abstract] OR badgers[Title/Abstract] OR hare[Title/Abstract] OR European hare[Title/Abstract] OR wild hares[Title/Abstract] OR North European hare[Title/Abstract] OR European brown hare[Title/Abstract] OR marten[Title/Abstract] OR racoon dogs[Title/Abstract] OR moles[Title/Abstract] OR rabbit[Title/Abstract] OR hamster[Title/Abstract] OR ferret[Title/Abstract] OR flies[Title/Abstract] OR wolves[Title/Abstract] OR ticks[Title/Abstract]) AND (spread of infection[Title/Abstract] OR spreading of microbes[Title/Abstract] OR spread of infectious agents[Title/Abstract] OR spread of pathogens[Title/Abstract] OR movement of pathogens[Title/Abstract] OR transfer of viruses[Title/Abstract] OR transfer of an infectious agent[Title/Abstract] OR transmission of pathogens[Title/Abstract] OR transmission of infectious pathogens[Title/Abstract] OR transmission[Title/Abstract] OR chain of Infection[Title/Abstract] OR circulation of infection[Title/Abstract] OR routes of infection[Title/Abstract] OR vectors of infection[Title/Abstract] OR vectors[Title/Abstract] OR transmitted by vectors[Title/Abstract] OR transmission of diseases[Title/Abstract] OR carries a disease[Title/Abstract] OR carry out a disease[Title/Abstract] OR biological vector[Title/Abstract] OR biological vectors[Title/Abstract] OR disease vector[Title/Abstract] OR disease vectors[Title/Abstract] OR mechanical vector[Title/Abstract] OR mechanical vectors[Title/Abstract] OR direct contact[Title/Abstract] OR fomites[Title/Abstract] OR vector borne[Title/Abstract] OR indirect contact[Title/Abstract] OR foodborne[Title/Abstract] OR vehicle borne[Title/Abstract]) AND (African swine fever[Title/Abstract] OR ASF*[Title/Abstract] OR classical swine fever[Title/Abstract] OR CSF[Title/Abstract] OR hog cholera[Title/Abstract] OR pig plague[Title/Abstract] OR Salmone*[Title/Abstract] OR Salmonella spp.[Title/Abstract] OR Salmonella[Title/Abstract] OR Aujeszky's disease[Title/Abstract] OR Pseudorabies[Title/Abstract] OR PRV[Title/Abstract] OR Brucella suis[Title/Abstract] OR swine brucellosis[Title/Abstract] OR Brucellossis in pigs[Title/Abstract] OR Brucella[Title/Abstract] OR Influenza[Title/Abstract] OR Porcine Influenza[Title/Abstract] OR influenza type A[Title/Abstract] OR FMD[Title/Abstract] OR Foot-and-Mouth Disease[Title/Abstract] OR Leptospirosis[Title/Abstract] OR Weil's disease[Title/Abstract] OR Leptospira interrogans[Title/Abstract] OR Leptospira borgpeterseni[Title/Abstract] OR Leptospira bratislava[Title/Abstract] OR Leptospira icterohaemorrhagiae[Title/Abstract] OR Trichinellosis[Title/Abstract] OR Trichinella spiralis[Title/Abstract] OR Trichinella britova[Title/Abstract] OR Trichinella spp.[Title/Abstract] OR Trichinella[Title/Abstract] OR HEV[Title/Abstract] OR Hepatitis

E[Title/Abstract] OR Rabies virus[Title/Abstract] OR RAB[Title/Abstract] OR Tuberculosis[Title/Abstract] OR Mycobacterium avium[Title/Abstract] OR avian tuberculosis[Title/Abstract] OR Mycobacterium tuberculosis[Title/Abstract] OR "Mycobacterium bovis[Title/Abstract] OR Transmissible gastroenteritis[Title/Abstract] OR TGE[Title/Abstract] OR Swine Erysipelas[Title/Abstract] OR Erysipelothrix rhusiopathiae[Title/Abstract] OR Erysipela[Title/Abstract] OR PED[Title/Abstract] OR Porcine epidemic diarrhoea virus[Title/Abstract] OR Porcine epidemic diarrhea[Title/Abstract] OR PRRS[Title/Abstract] OR Porcine reproductive and respiratory syndrome[Title/Abstract] OR swine dysentery[Title/Abstract] OR Brachyspira hyodysenteriae[Title/Abstract] OR B. pilosicoli[Title/Abstract] OR Encephalomyocarditis[Title/Abstract] OR EMCV[Title/Abstract] OR virus of encephalomyocarditis[Title/Abstract] OR atrophic rhinitis[Title/Abstract] OR Pasteurella multocida[Title/Abstract] OR B. bronchiseptica[Title/Abstract] OR enzootic pneumonia[Title/Abstract] OR "Mycoplasmal pneumonia[Title/Abstract] OR Mycoplasma hyopneumoniae[Title/Abstract] OR dermatophytes[Title/Abstract] OR Trichophytum mentagrophytes[Title/Abstract] OR Streptococcal infections[Title/Abstract] OR Streptococcus suis[Title/Abstract] OR Streptococcus equisimilis[Title/Abstract] OR "Streptococcus porcinus[Title/Abstract] OR porcine proliferative enteropathy[Title/Abstract] OR Lawsonia intracellularis bacterium[Title/Abstract] OR yersiniosis[Title/Abstract] OR Yersinia pseudotuberculosis[Title/Abstract] OR Y. enterocolitica[Title/Abstract] OR campylobacteriosis[Title/Abstract] OR Campylobacter jejuni[Title/Abstract] OR Campylobacter hyointestinalis[Title/Abstract] OR Campylobacter mucosalis[Title/Abstract] OR Campylobacter coli[Title/Abstract] OR Actinobacillus equuli[Title/Abstract] OR Actinobacillus suis[Title/Abstract] OR Actinobacillosis[Title/Abstract] OR Toxoplasmosis[Title/Abstract] OR Toxoplasma gondii[Title/Abstract] OR Cryptosporidium suis[Title/Abstract] OR Cryptosporidiosis[Title/Abstract] OR Cryptosporidium parvum[Title/Abstract] OR giardiosis[Title/Abstract] OR Giardia intestinalis[Title/Abstract] OR Methicillin-resistant Staphylococcus aureus infection[Title/Abstract] OR staphylococcus aureus[Title/Abstract] OR MRSA[Title/Abstract] OR Bordetellosis[Title/Abstract] OR Bordetella bronchiseptica[Title/Abstract] OR Haemophilus parasuis[Title/Abstract] OR Colibacillosis[Title/Abstract] OR Escherichia coli[Title/Abstract] OR Glasser disease[Title/Abstract] OR Glaesserella parasuis[Title/Abstract] OR Porcine circovirosis type 2[Title/Abstract] OR PCV2[Title/Abstract] OR Swine vesicular disease[Title/Abstract] OR Swine vesicular disease virus[Title/Abstract] OR EVP[Title/Abstract]) AND (Europe[Title/Abstract] OR European countries[Title/Abstract] OR EU countries[Title/Abstract] OR EU country[Title/Abstract] OR EU member[Title/Abstract] OR non-EU country[Title/Abstract] OR Albania[Title/Abstract] OR Andorra[Title/Abstract] OR Austria[Title/Abstract] OR Belarus[Title/Abstract] OR Belgium[Title/Abstract] OR Bosnia and Herzegovina[Title/Abstract] OR Bulgaria[Title/Abstract] OR Croatia[Title/Abstract] OR Republic of Cyprus[Title/Abstract] OR Czech Republic[Title/Abstract] OR Denmark[Title/Abstract] OR Estonia[Title/Abstract] OR Finland[Title/Abstract] OR France[Title/Abstract] OR Germany[Title/Abstract] OR Greece[Title/Abstract] OR Holy See[Title/Abstract] OR Hungary[Title/Abstract] OR Iceland[Title/Abstract] OR Ireland[Title/Abstract] OR Italy[Title/Abstract] OR Latvia[Title/Abstract] OR Liechtenstein[Title/Abstract] OR Lithuania[Title/Abstract] OR Luxembourg[Title/Abstract] OR Malta[Title/Abstract] OR Moldova[Title/Abstract] OR Monaco[Title/Abstract] OR Montenegro[Title/Abstract] OR Netherlands[Title/Abstract] OR North Macedonia[Title/Abstract] OR Norway[Title/Abstract] OR Poland[Title/Abstract] OR Portugal[Title/Abstract] OR Romania[Title/Abstract] OR San Marino[Title/Abstract] OR Serbia[Title/Abstract] OR Slovakia[Title/Abstract] OR Slovenia[Title/Abstract] OR Spain[Title/Abstract] OR Sweden[Title/Abstract] OR Switzerland[Title/Abstract] OR Ukraine[Title/Abstract] OR United Kingdom[Title/Abstract])

Scopus

TITLE-ABS-KEY ("pig" OR "pigs" OR "farm pig" OR "farm pigs" OR "swine" OR "sow" OR "porcine" OR "hog " OR "commercial pigs" OR "commercial pigs farming" OR "commercial swine industry" OR "commercial swine production" OR "intensive pig farming" OR "intensive pig farms" OR "intensive pig production" OR "pig factory farming" OR "pig farming" OR "free range pig farming" OR "free-ranging pig holding" OR "free range pigs" OR "open air pig farming" OR "Outdoor Pig Production" **AND** "wild pig" OR "wild pigs" OR "wild boar" OR "wild boars" OR "wild swine" OR "wild hog" OR "feral pig" OR "feral pigs" OR "feral hog" OR "feral hogs" OR "Sus scrofa" OR "Old World swine" OR "feral swine" OR "wild birds" OR "aquatic birds" OR "waterfowl" OR "pigeons" OR "starlings" OR "ducks" OR "homeless dogs" OR "stray dogs" OR "stray dog" OR "cats" OR "waterfowl" OR "pigeons" OR "rodents" OR "rats" OR "mice" OR "house mouse" OR "Mus musculus" OR "brown rat*" OR "Norwegian rat" OR "Rattus norvegicus" OR "feral rodents" OR "black rats" OR "house mice" OR "barn rats" OR "wild rodents" OR "feral rodents" OR "rodents on farms" OR "mice" OR "deer" OR "red deer" OR "fallow deer" OR "badgers" OR "hare" OR "European hare" OR "wild hares" OR "North European hare" OR "European brown hare" OR "marten" OR "racoon dogs" OR "moles" OR "rabbit" OR "hamster" OR "ferret" OR "flies" OR "wolves" OR ticks **AND** "spread of infection" OR "spreading of microbes" OR "spread of infectious agents" OR "spread of pathogens" OR "movement of pathogens" OR "transfer of viruses" OR "transfer of an infectious agent" OR "transmission of pathogens" OR "transmission of infectious pathogens" OR transmission OR "chain of Infection" OR "circulation of infection" OR "routes of infection" OR "vectors of infection" OR vectors OR "transmitted by vectors" OR "transmission of diseases" OR "carries a disease" OR "carry out a disease" OR "biological vector" OR "biological vectors" OR "disease vector" OR "disease vectors" OR "mechanical vector" OR "mechanical vectors" OR "direct contact" OR fomites OR "vector borne" OR "indirect contact" OR foodborne OR "vehicle borne" **AND** "African swine fever" OR ASF OR ASFV OR "Classical Swine Fever" OR CSF OR "hog cholera" OR "pig plague" OR salmone* OR "Salmonella spp." OR Salmonella OR "Aujeszky's disease" OR Pseudorabies OR PRV OR "Brucella suis" OR "swine brucellosis" OR "Brucellossis in pigs" OR Brucella OR Influenza OR "Porcine Influenza" OR "influenza type A" OR "swine influenza virus" OR "FMD" OR "Foot-and-Mouth Disease" OR "FMDV" OR "Leptospirosis" OR "Weil's disease" OR "Leptospira interrogans" OR "Leptospira borgpeterseni" OR "Leptospira bratislava" OR "Leptospira icterohaemorrhagiae" OR Trichinellosis OR "Trichinella spiralis" OR "Trichinella britova" OR "Trichinella spp" OR Trichinella OR HEV OR "Hepatitis E" OR "Rabies virus" OR RABV OR Tuberculosis OR "Mycobacterium avium" OR "avian tuberculosis" OR "Mycobacterium tuberculosis" OR "Mycobacterium bovis" OR "Transmissible gastroenteritis" OR TGE OR "Swine Erysipelas" OR "Erysipelothrix rhusiopathiae" OR Erysipela OR PED OR "Porcine epidemic diarrhoea virus" OR "Porcine epidemic diarrhea" OR PRRS OR "Porcine reproductive and respiratory syndrome " OR "swine dysentery" OR "Brachyspira hyodysenteriae" OR "B. pilosicoli" OR Encephalomyocarditis OR EMCV OR "virus of encephalomyocarditis" OR "atrophic rhinitis" OR "Pasteurella multocida" OR "B. bronchiseptica" OR "enzootic pneumonia" OR "Mycoplasmal pneumonia" OR "Mycoplasma hyopneumoniae" OR dermatophytes OR "Trichophytum mentagrophytes" OR "Streptococcal infections" OR "Streptococcus suis" OR "Streptococcus equisimilis" OR "Streptococcus

porcinus" OR "porcine proliferative enteropathy" OR "Lawsonia intracellularis bacterium" OR "yersiniosis" OR "Yersinia pseudotuberculosis" OR "Y. enterocolitica" OR campylobacteriosis OR "Campylobacter jejuni" OR "Campylobacter hyointestinalis" OR "Campylobacter mucosalis" OR "Campylobacter coli" OR "Actinobacillus equuli" OR "Actinobacillus suis" OR Actinobacillosis OR Toxoplasmosis OR "Toxoplasma gondii" OR "Cryptosporidium suis" OR Cryptosporidiosis OR "Cryptosporidium parvum" OR giardiosis OR "Giardia intestinalis" OR "Methicillin-resistant Staphylococcus aureus infection" OR "staphylococcus aureus" OR MRSA OR Bordetellosis OR " Bordetella bronchiseptica" OR "Haemophilus parasuis" OR Colibacillosis OR "Escherichia coli" OR "Glasser disease" OR "Glaesserella parasuis" OR "Porcine circovirosis type 2" OR PCV2 OR "Swine vesicular disease" OR "Swine vesicular disease virus" OR EVP **AND** "Europe" OR "European countries" OR "EU countries" OR "EU country" OR "EU member" OR "non-EU country" OR Albania OR Andorra OR Austria OR Belarus OR Belgium OR "Bosnia and Herzegovina" OR Bulgaria OR Croatia OR "Republic of Cyprus" OR "Czech Republic" OR Denmark OR Estonia OR Finland OR France OR Germany OR Greece OR "Holy See" OR Hungary OR Iceland OR Ireland OR Italy OR Latvia OR Liechtenstein OR Lithuania OR Luxembourg OR Malta OR Moldova OR Monaco OR Montenegro OR Netherlands OR "North Macedonia" OR Norway OR Poland OR Portugal OR Romania OR "San Marino" OR Serbia OR Slovakia OR Slovenia OR Spain OR Sweden OR Switzerland OR Ukraine OR "United Kingdom")

WoS

AB=((pig OR pigs OR "farm pig" OR "farm pigs" OR swine OR sow OR porcine OR hog OR "commercial pigs" OR "commercial pigs farming" OR "commercial swine industry" OR "commercial swine production" OR "intensive pig farming" OR "intensive pig farms" OR "intensive pig production" OR "pig factory farming" OR "pig farming" OR "free range pig farming" OR "free-ranging pig holding" OR "free range pigs") AND ("wild pig" OR "wild pigs" OR "wild boar" OR "wild boars" OR "wild swine" OR "wild hog" OR "feral pig" OR "feral pigs" OR "feral hog" OR "feral hogs" OR "Sus scrofa" OR "Old World swine" OR "feral swine" OR "wild birds" OR "aquatic birds" OR "waterfowl" OR "pigeons" OR "starlings" OR "ducks" OR "homeless dogs" OR "stray dogs" OR "stray dog" OR "cats" OR "waterfowl" OR "pigeons" OR "rodents" OR "rats" OR "mice" OR "house mouse" OR "Mus musculus" OR "brown rat*" OR "Norwegian rat" OR "Rattus norvegicus" OR "feral rodents" OR "black rats" OR "house mice" OR "barn rats" OR "wild rodents" OR "feral rodents" OR "rodents on farms" OR "mice" OR "deer" OR "red deer" OR "fallow deer" OR "badgers" OR "hare" OR "European hare" OR "wild hares" OR "North European hare" OR "European brown hare" OR "marten" OR "racoon dogs" OR "moles" OR "rabbit" OR "hamster" OR "ferret" OR "flies" OR "wolves" OR ticks) AND ("spread of infection" OR "spreading of microbes" OR "spread of infectious agents" OR "spread of pathogens" OR "movement of pathogens" OR "transfer of viruses" OR "transfer of an infectious agent" OR "transmission of pathogens" OR "transmission of infectious pathogens" OR transmission OR "chain of Infection" OR "circulation of infection" OR "routes of infection" OR "vectors of infection" OR vectors OR "transmitted by vectors" OR "transmission of diseases" OR "carries a disease" OR "carry out a disease" OR "biological vector" OR "biological vectors" OR "disease vector" OR "disease vectors" OR "mechanical vector" OR "mechanical vectors" OR "direct contact" OR fomites OR "vector borne" OR "indirect contact" OR foodborne OR "vehicle borne")

AND ("African swine fever" OR ASF OR ASFV OR "Classical Swine Fever" OR CSF OR "hog cholera" OR "pig plague" OR salmone* OR "Salmonella spp." OR Salmonella OR "Aujeszky's disease" OR Pseudorabies OR PRV OR "Brucella suis" OR "swine brucellosis" OR "Brucellossis in pigs" OR Brucella OR Influenza OR "Porcine Influenza" OR "influenza type A" OR "FMD" OR "Foot-and-Mouth Disease" OR "FMDV" OR "Leptospirosis" OR "Weil's disease" OR "Leptospira interrogans" OR "Leptospira borgpeterseni" OR "Leptospira bratislava" OR "Leptospira icterohaemorrhagiae" OR Trichinellosis OR "Trichinella spiralis" OR "Trichinella britova" OR "Trichinella spp" OR Trichinella OR HEV OR "Hepatitis E" OR "Rabies virus" OR RABV OR Tuberculosis OR "Mycobacterium avium" OR "avian tuberculosis" OR "Mycobacterium tuberculosis" OR "Mycobacterium bovis" OR "Transmissible gastroenteritis" OR TGE OR "Swine Erysipelas" OR "Erysipelothrix rhusiopathiae" OR Erysipela OR PED OR "Porcine epidemic diarrhoea virus" OR "Porcine epidemic diarrhea" OR PRRS OR "Porcine reproductive and respiratory syndrome " OR "swine dysentery" OR "Brachyspira hyodysenteriae" OR "B. pilosicoli" OR Encephalomyocarditis OR EMCV OR "virus of encephalomyocarditis" OR "atrophic rhinitis" OR "Pasteurella multocida" OR "B. bronchiseptica" OR "enzootic pneumonia" OR "Mycoplasmal pneumonia" OR "Mycoplasma hyopneumoniae" OR dermatophytes OR "Trichophytum mentagrophytes" OR "Streptococcal infections" OR "Streptococcus suis" OR "Streptococcus equisimilis" OR "Streptococcus porcinus" OR "porcine proliferative enteropathy" OR "Lawsonia intracellularis bacterium" OR "yersiniosis" OR "Yersinia pseudotuberculosis" OR "Y. enterocolitica" OR campylobacteriosis OR "Campylobacter jejuni" OR "Campylobacter hyointestinalis" OR "Campylobacter mucosalis" OR "Campylobacter coli" OR "Actinobacillus equuli" OR "Actinobacillus suis" OR Actinobacillosis OR Toxoplasmosis OR "Toxoplasma gondii" OR "Cryptosporidium suis" OR Cryptosporidiosis OR "Cryptosporidium parvum" OR giardiosis OR "Giardia intestinalis" OR "Methicillin-resistant Staphylococcus aureus infection" OR "staphylococcus aureus" OR MRSA OR Bordetellosis OR " Bordetella bronchiseptica" OR "Haemophilus parasuis" OR Colibacillosis OR "Escherichia coli" OR "Glasser disease" OR "Glaesserella parasuis" OR "Porcine circovirosis type 2" OR PCV2 OR "Swine vesicular disease" OR "Swine vesicular disease virus" OR EVP) AND (Europe OR "European countries" OR "EU countries" OR "EU country" OR "EU member" OR "non-EU country" OR Albania OR Andorra OR Austria OR Belarus OR Belgium OR "Bosnia and Herzegovina" OR Bulgaria OR Croatia OR "Republic of Cyprus" OR "Czech Republic" OR Denmark OR Estonia OR Finland OR France OR Germany OR Greece OR "Holy See" OR Hungary OR Iceland OR Ireland OR Italy OR Latvia OR Liechtenstein OR Lithuania OR Luxembourg OR Malta OR Moldova OR Monaco OR Montenegro OR Netherlands OR "North Macedonia" OR Norway OR Poland OR Portugal OR Romania OR "San Marino" OR Serbia OR Slovakia OR Slovenia OR Spain OR Sweden OR Switzerland OR Ukraine OR "United Kingdom"))

Supplementary Table S3. Direct or indirect pathogen transmission pathways* from wildlife and pests to domestic pigs.

Pathogen	Direct contact with wild boars	People	Manure	Wild animals (non-wild boars)/wild birds	Rodents	Insects/ticks (Vectors)	Aerosol	Animal feed	Water	Fomites
**Viral pathogens**										
**African swine fever virus (ASFV)**	[27], [37], [56], [58], [76], [78], [86], [100],	[27], [37], [100]		[27], [100]	[27]	[58], [75]	[100]	[27], [76], [100]	[27], [100]	[27], [37], [60], [100]
**Aujeszky's disease virus (ADV)**	[42], [46], [87], [89], [88]	[89]					[88,89]			[88,89]
**Foot and mouth disease virus (FMDV)**	[28]									
**Hepatitis E virus (HEV)**	[25],[29], [31], [39], [50], [51]		[49]		[31], [49]			[51]	[51]	
**Porcine circovirus type 2, 3 (PCV-2, 3)**	[24], [54], [97], [108]	[24]								
**Porcine epidemic diarrhoea virus (PEDV)**	[55] [68]									
**Porcine parvovirus, type 2, 3 (PPV-2, 3),**	[82], [97]									
**Porcine reproductive and respiratory syndrome virus (PRRSV)**	[20], [61], [95], [101,102]									
**Swine influenza virus (SIV)**	[97]									
**Transmissible gastroenteritis virus (TGEV)**	[55]									
**Bacterial pathogens**										
Brachyspira hampsonii				[91]						
Brucella suis	[39], [47], [48], [62], [75], [79], [90], [101,102]	[26]		[26], [90]						
Erysipelothri rhusiopathiae	[46], [97]									
Escherichia coli	[40], [41],[99]							[40]	[40]	
***Leptospira* spp.**	[52]				[63]					
Listeria monocytogenes	[99]									
**Methicillin-resistant *Staphylococcus aureus* (MRSA)**						[35]				
Mycoplasma hyopneumoniae	[46], [97], [103]	[103]					[103]			[103]

***Salmonella* spp.**	[39], [84], [85], [99], [105],		[106]	[106]						
Mycobacterium tuberculosis, bovis	[93], [94], [107]			[53,92–94,107]					[94]	
***Yersinia* spp.**	[99]				[98]					
**Parasites**										
Toxoplasma gondii	[39], [69], [97]			[96]	[36], [63]					
***Trichinella* spp.**	[39], [70] [81], [83]			[70],[80], [83]	[70]			[83]		

* In the present table, we included the information from the selected studies, however, there could be other possible transmission pathways.
